# Supplementary figures and images for: Identifying Hosts of Families of Viruses: A Machine Learning Approach
Source: PLoS One. 2011 Dec 9;6(12):e27631. doi: 10.1371/journal.pone.0027631 (PMC3235098; doi:10.1371/journal.pone.0027631)

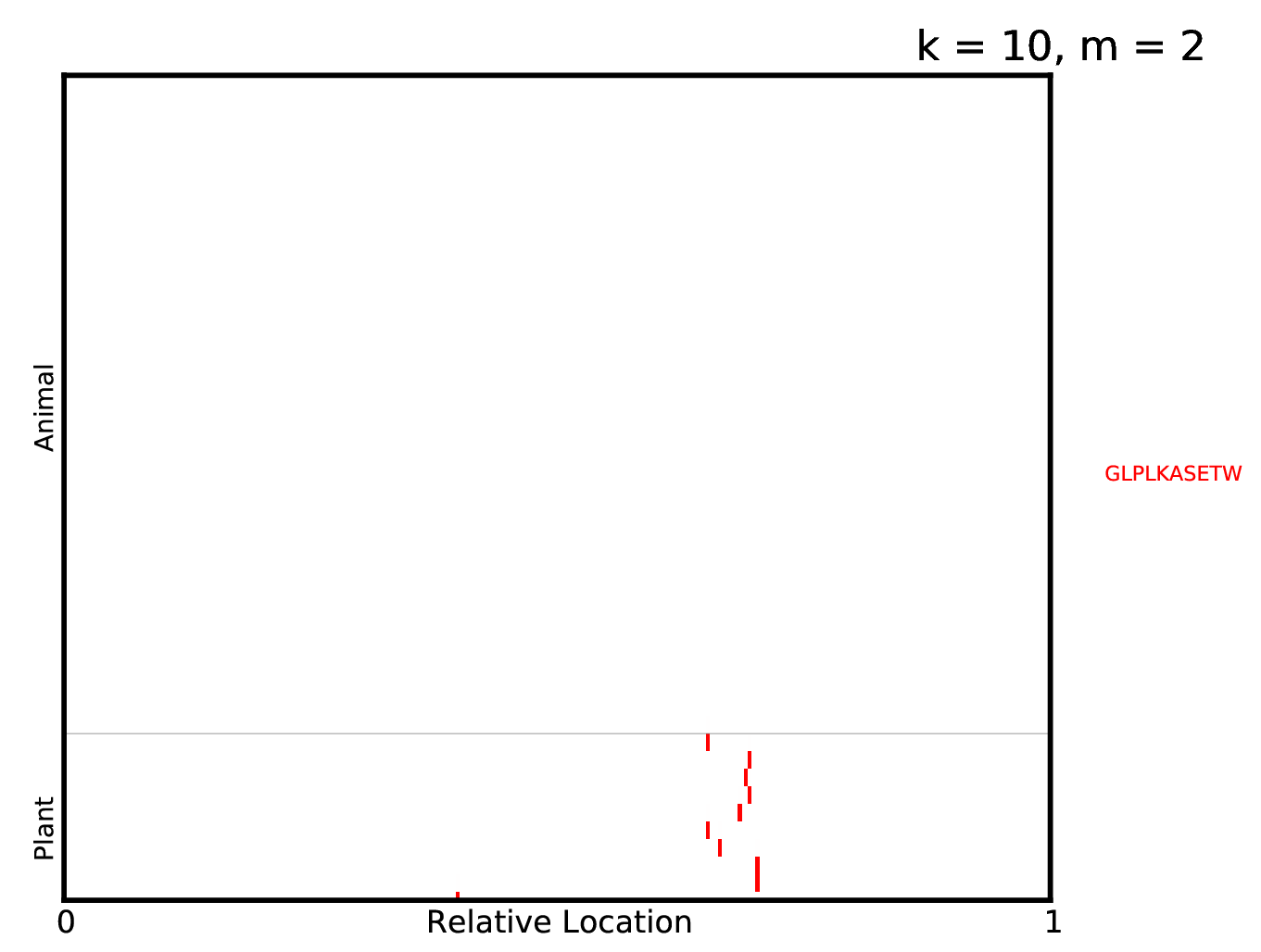

Supplement: Figure S1 — Visualizing predictive subsequences for Rhabdoviridae . A visualization of the mismatch neighborhood of the -mer selected in an ADT for Rhabdoviridae, where . The virus proteomes are grouped vertically by their label with their lengths scaled to . Regions containing elements of the mismatch neighborhood of each -mer are then indicated on the virus proteome. Note that, for Rhabdoviridae, plant and animal viruses could be distinguished with just one -mer. (TIFF) [file pone.0027631.s001.tiff]
