# Supplementary material for: Identifying Hosts of Families of Viruses: A Machine Learning Approach
Source: PLoS One. 2011 Dec 9;6(12):e27631. doi: 10.1371/journal.pone.0027631 (PMC3235098; doi:10.1371/journal.pone.0027631)
Supplement: Table S1 — List of viruses in Picornaviridae family used in learning. (PDF) [file pone.0027631.s003.pdf]

Table S 1: List of viruses in *Picornaviridae* family used in learning.

Identifier	Name	Host	Subfamily
NC_014137	Honey bee slow paralysis virus	Invertebrate	Cripavirus
NC_001366	Theilovirus	Vertebrate	Cardiovirus
NC_003005	Taura syndrome virus	Invertebrate	Cripavirus
NC_003783	Triatoma virus	Invertebrate	Cripavirus
NC_003077	Equine rhinitis B virus 2	Vertebrate	Erbovirus
NC_001834	Drosophila C virus	Invertebrate	Cripavirus
NC_003784	Black queen cell virus	Invertebrate	Cripavirus
NC_002066	Sacbrood virus	Invertebrate	Iflavirus
NC_008182	Black raspberry necrosis virus RNA1	Plant	Cripavirus
NC_013115	Human enterovirus 107	Vertebrate	Enterovirus
NC_013114	Human enterovirus 98	Vertebrate	Enterovirus
NC_005092	Ectropis obliqua picorna-like virus	Invertebrate	unassigned
NC_013695	Simian picornavirus strain N203	Vertebrate	Enterovirus
NC_010384	Simian picornavirus strain N125	Vertebrate	Enterovirus
NC_008183	Black raspberry necrosis virus RNA2	Plant	Cripavirus
NC_011829	Porcine kobuvirus swine/S-1-HUN/2007/Hungary	Vertebrate	Kobuvirus
NC_013755	Kobuvirus pig/JY-2010a/CHN	Vertebrate	Kobuvirus
NC_012986	Human klassevirus 1	Vertebrate	unassigned
NC_012957	Salivirus NG-J1	Vertebrate	unassigned
NC_011190	Mikania micrantha mosaic virus RNA1	Plant	Cripavirus
NC_011189	Mikania micrantha mosaic virus RNA2	Plant	Cripavirus
NC_010354	Bovine rhinitis B virus	Vertebrate	Erbovirus
NC_008250	Duck hepatitis A virus	Vertebrate	Hepatovirus
NC_006553	Avian sapelovirus	Vertebrate	Sapelovirus
NC_013219	Turnip ringspot virus RNA 2	Plant	Cripavirus
NC_013218	Turnip ringspot virus RNA 1	Plant	Cripavirus
NC_011451	Foot-and-mouth disease virus - type SAT 1	Vertebrate	Aphthovirus
NC_011450	Foot-and-mouth disease virus - type A	Vertebrate	Aphthovirus
NC_005266	Raspberry ringspot virus RNA1	Plant	Cripavirus
NC_003992	Foot-and-mouth disease virus - type SAT 2	Vertebrate	Aphthovirus
NC_004915	Foot-and-mouth disease virus - type Asia 1	Vertebrate	Aphthovirus
NC_004807	Kashmir bee virus	Invertebrate	Cripavirus
NC_004451	Simian picornavirus 1	Vertebrate	unassigned
NC_004365	Aphid lethal paralysis virus	Invertebrate	Cripavirus
NC_004004	Foot-and-mouth disease virus - type O	Vertebrate	Aphthovirus
NC_003987	Porcine enterovirus 8	Vertebrate	Enterovirus
NC_003924	Cricket paralysis virus	Invertebrate	Cripavirus
NC_003113	Perina nuda virus	Invertebrate	Iflavirus
NC_002554	Foot-and-mouth disease virus - type C	Vertebrate	Aphthovirus
NC_001874	Rhopalosiphum padi virus	Invertebrate	Cripavirus
NC_001490	Human rhinovirus 14	Vertebrate	Rhinovirus
NC_001479	Encephalomyocarditis virus	Vertebrate	Cardiovirus
NC_012212	Chaetoceros socialis f. radians RNA virus segment 1	Plant	Cripavirus
NC_012802	Human cosavirus D1	Vertebrate	unassigned
NC_012801	Human cosavirus B1	Vertebrate	unassigned
NC_012800	Human cosavirus A1	Vertebrate	unassigned
NC_012798	Human cosavirus E1	Vertebrate	unassigned
NC_010411	Simian picornavirus 17	Vertebrate	unassigned
NC_003446	Strawberry mottle virus RNA 2	Plant	Cripavirus
NC_003445	Strawberry mottle virus RNA 1	Plant	Cripavirus
NC_010415	Simian enterovirus SV6	Vertebrate	Enterovirus

NC_010413	Simian enterovirus SV43	Vertebrate	Enterovirus
NC_010412	Simian enterovirus SV19	Vertebrate	Enterovirus
NC_003792	Cycas necrotic stunt virus RNA 2	Plant	Cripavirus
NC_003791	Cycas necrotic stunt virus RNA 1	Plant	Cripavirus
NC_010988	Tomato marchitez virus RNA 2	Plant	Cripavirus
NC_010987	Tomato marchitez virus RNA 1	Plant	Cripavirus
NC_009891	Seal picornavirus type 1	Vertebrate	unassigned
NC_009758	Marine RNA virus JP-B	Plant	unassigned
NC_009757	Marine RNA virus JP-A	Plant	unassigned
NC_009530	Brevicoryne brassicae picorna-like virus	Invertebrate	unassigned
NC_009448	Saffold virus	Vertebrate	Cardiovirus
NC_009032	Tomato torrado virus RNA2	Plant	Cripavirus
NC_009013	Tomato torrado virus RNA1	Plant	Cripavirus
NC_006964	Strawberry latent ringspot virus RNA1	Plant	Cripavirus
NC_005281	Heterosigma akashiwo RNA virus SOG263	Plant	Marnavirus
NC_005097	Tobacco ringspot virus RNA 1	Plant	Cripavirus
NC_005096	Tobacco ringspot virus RNA 2	Plant	Cripavirus
NC_004439	Tomato black ring virus RNA 1	Plant	Cripavirus
NC_004421	Bovine kobuvirus	Vertebrate	Kobuvirus
NC_003988	Simian enterovirus A	Vertebrate	Enterovirus
NC_003983	Equine rhinitis B virus 1	Vertebrate	Erbovirus
NC_003974	Patchouli mild mosaic virus RNA 2	Plant	Cripavirus
NC_003840	Tomato ringspot virus RNA 1	Plant	Cripavirus
NC_003839	Tomato ringspot virus RNA 2	Plant	Cripavirus
NC_003788	Apple latent spherical virus segment 2	Plant	Cripavirus
NC_003787	Apple latent spherical virus segment 1	Plant	Cripavirus
NC_003741	Red clover mottle virus RNA 1	Plant	Cripavirus
NC_003738	Red clover mottle virus RNA 2	Plant	Cripavirus
NC_003694	Beet ringspot virus RNA 2	Plant	Cripavirus
NC_003693	Beet ringspot virus RNA 1	Plant	Cripavirus
NC_003622	Grapevine chrome mosaic virus RNA 1	Plant	Cripavirus
NC_003621	Grapevine chrome mosaic virus RNA 2	Plant	Cripavirus
NC_003615	Grapevine fanleaf virus RNA 1	Plant	Cripavirus
NC_003550	Cowpea mosaic virus RNA 2	Plant	Cripavirus
NC_003549	Cowpea mosaic virus RNA 1	Plant	Cripavirus
NC_003509	Blackcurrant reversion virus RNA1	Plant	Cripavirus
NC_003502	Blackcurrant reversion virus RNA 2	Plant	Cripavirus
NC_003545	Cowpea severe mosaic virus RNA 1	Plant	Cripavirus
NC_003544	Cowpea severe mosaic virus RNA 2	Plant	Cripavirus
NC_003975	Patchouli mild mosaic virus RNA 1	Plant	Cripavirus
NC_010710	Radish mosaic virus RNA2	Plant	Cripavirus
NC_010709	Radish mosaic virus RNA1	Plant	Cripavirus
NC_009996	Human rhinovirus C	Vertebrate	Rhinovirus
NC_009887	Human enterovirus 100	Vertebrate	Enterovirus
NC_009750	Duck hepatitis virus AP	Vertebrate	Hepatovirus
NC_009025	Israel acute paralysis virus of bees	Invertebrate	Cripavirus
NC_006965	Strawberry latent ringspot virus RNA2	Plant	Cripavirus
NC_006272	Cherry rasp leaf virus RNA2	Plant	Cripavirus
NC_006271	Cherry rasp leaf virus	Plant	Cripavirus
NC_006057	Arabis mosaic virus RNA 1	Plant	Cripavirus
NC_006056	Arabis mosaic virus RNA 2	Plant	Cripavirus
NC_005290	Broad bean wilt virus 1 RNA 2	Plant	Cripavirus
NC_005289	Broad bean wilt virus 1 RNA 1	Plant	Cripavirus
NC_005267	Raspberry ringspot virus RNA 2	Plant	Cripavirus

NC_004830	Deformed wing virus	Invertebrate	Cripavirus
NC_004441	Porcine enterovirus B	Vertebrate	Enterovirus
NC_004440	Tomato black ring virus RNA 2	Plant	Cripavirus
NC_003990	Avian encephalomyelitis virus	Vertebrate	Tremovirus
NC_003985	Porcine teschovirus 1	Vertebrate	Teschovirus
NC_003982	Equine rhinitis A virus	Vertebrate	Aphthovirus
NC_003976	Ljungan virus	Vertebrate	Parechovirus
NC_003800	Squash mosaic virus RNA 2	Plant	Cripavirus
NC_003799	Squash mosaic virus RNA 1	Plant	Cripavirus
NC_003782	Himetobi P virus	Invertebrate	Cripavirus
NC_003781	Infectious flacherie virus	Invertebrate	Iflavirus
NC_003628	Parsnip yellow fleck virus	Plant	Sequivirus
NC_003626	Maize chlorotic dwarf virus	Plant	Waikivirus
NC_003623	Grapevine fanleaf virus RNA 2	Plant	Cripavirus
NC_003496	Bean pod mottle virus RNA 1	Plant	Cripavirus
NC_003495	Bean pod mottle virus RNA 2	Plant	Cripavirus
NC_003004	Broad bean wilt virus 2 RNA2	Plant	Cripavirus
NC_003003	Broad bean wilt virus 2 RNA1	Plant	Cripavirus
NC_002548	Acute bee paralysis virus	Invertebrate	Cripavirus
NC_001918	Aichi virus	Vertebrate	Kobuvirus
NC_001897	Human parechovirus	Vertebrate	Parechovirus
NC_001859	Bovine enterovirus	Vertebrate	Enterovirus
NC_001632	Rice tungro spherical virus	Plant	Waikavirus
NC_001617	Human rhinovirus 89	Vertebrate	Rhinovirus
NC_001612	Human enterovirus A	Vertebrate	Enterovirus
NC_002058	Poliovirus	Vertebrate	Enterovirus
NC_001489	Hepatitis A virus	Vertebrate	Parechovirus
NC_001472	Human enterovirus B	Vertebrate	Enterovirus
NC_001430	Human enterovirus D	Vertebrate	Enterovirus
NC_001428	Human enterovirus C	Vertebrate	Enterovirus
NC_005876	Kakugo virus	Invertebrate	Iflavirus
NC_003779	Plautia stali intestine virus	Invertebrate	Cripavirus
NC_010810	Human TMEV-like cardiovirus	Vertebrate	Cardiovirus
NC_011349	Seneca valley virus	Vertebrate	Senecavirus
NC_007522	Schizochytrium single-stranded RNA virus	Plant	unassigned
NC_006559	Solenopsis invicta virus 1	Invertebrate	Cripavirus
NC_003785	Satsuma dwarf virus RNA 1	Plant	Cripavirus
NC_003786	Satsuma dwarf virus RNA 2	Plant	Cripavirus
NC_008029	Homalodisca coagulata virus-1	Invertebrate	Cripavirus
