# Supplementary material for: Identifying Hosts of Families of Viruses: A Machine Learning Approach
Source: PLoS One. 2011 Dec 9;6(12):e27631. doi: 10.1371/journal.pone.0027631 (PMC3235098; doi:10.1371/journal.pone.0027631)
Supplement: Table S2 — List of viruses in Rhabdoviridae family used in learning. (PDF) [file pone.0027631.s004.pdf]

Table S 2: List of viruses in *Rhabdoviridae* family used in learning.

Identifier	Name	Host	Subfamily
NC_009609	Orchid fleck virus RNA 2	Plant	unassigned
NC_006942	Taro vein chlorosis virus	Plant	Nucleorhabdovirus
NC_005975	Maize mosaic virus	Plant	Nucleorhabdovirus
EF614258	West Caucasian bat virus	Animal	Lyssavirus
DQ186554	Iranian maize mosaic nucleorhabdovirus	Plant	Nucleorhabdovirus
NC_007642	Lettuce necrotic yellows virus	Plant	Cytorhabdovirus
EF687738	Lettuce yellow mottle virus	Plant	Cytorhabdovirus
NC_002251	Northern cereal mosaic virus	Plant	Cytorhabdovirus
NC_001615	Sonchus yellow net virus	Plant	Cytorhabdovirus
NC_005974	Maize fine streak virus	Plant	Nucleorhabdovirus
NC_003746	Rice yellow stunt virus	Plant	Nucleorhabdovirus
DQ491000	Spring viremia of carp virus isolate A2	Animal	Vesiculovirus
EU373657	Cocal virus Indiana 2	Animal	Vesiculovirus
AJ318079	Spring Viremia of Carp	Animal	Vesiculovirus
NC_002803	Spring viremia of carp virus	Animal	Vesiculovirus
AF104985	Hirame rhabdovirus strain CA 9703	Animal	Novirhabdovirus
NC_005093	Hirame rhabdovirus	Animal	Novirhabdovirus
EU177782	Spring viremia of carp virus isolate BJ0505-2	Animal	Vesiculovirus
EU373658	Vesicular stomatitis Alagoas virus Indiana 3	Animal	Vesiculovirus
AJ810084	Isfahan virus N gene	Animal	Vesiculovirus
DQ097384	Spring viremia of carp virus isolate A1	Animal	Vesiculovirus
NC_001652	Infectious hematopoietic necrosis virus	Animal	Novirhabdovirus
X89213	Infectious haematopoietic necrosis virus (IHNV)	Animal	Novirhabdovirus
NC_000855	Viral hemorrhagic septicemia virus	Animal	Novirhabdovirus
Y18263	Viral hemorrhagic septicemia virus strain Fil3 RNA	Animal	Novirhabdovirus
AY840978	Tupaia rhabdovirus	Animal	Dimarhabdovirus
NC_007020	Tupaia rhabdovirus	Animal	Dimarhabdovirus
NC_008514	Siniperca chuatsi rhabdovirus	Animal	Dimarhabdovirus
DQ399789	Siniperca chuatsi rhabdovirus from China	Animal	Dimarhabdovirus
AF147498	Snakehead rhabdovirus	Animal	Novirhabdovirus
NC_000903	Snakehead rhabdovirus	Animal	Novirhabdovirus
AF081020	Australian bat lyssavirus	Animal	Lyssavirus
EF614261	Khujand lyssavirus	Animal	Lyssavirus
EF614259	Aravan virus	Animal	Lyssavirus
AF418014	Australian bat lyssavirus	Animal	Lyssavirus
EU293116	Rabies virus isolate 9704ARG	Animal	Lyssavirus
EU293115	Rabies virus isolate 9147FRA	Animal	Lyssavirus
EU293121	Rabies virus isolate 8743THA	Animal	Lyssavirus
EU293114	European bat lyssavirus 2 isolate 9018HOL	Animal	Lyssavirus
NC_009528	European bat lyssavirus 2	Animal	Lyssavirus
NC_001542	Rabies virus	Animal	Lyssavirus
NC_006429	Mokola virus	Animal	Lyssavirus
EU293117	Mokola virus isolate 86100CAM	Animal	Lyssavirus
EU293118	Mokola virus isolate 86101RCA	Animal	Lyssavirus
NC_009527	European bat lyssavirus 1	Animal	Lyssavirus
EF614260	Irkut virus	Animal	Lyssavirus
EU293110	Lagos bat virus isolate 8619NGA	Animal	Lyssavirus
EU293108	Lagos bat virus isolate 0406SEN	Animal	Lyssavirus
NC_002526	Bovine ephemeral fever virus	Animal	Ephemerovirus
